# Trend of passive smoking and associated factors in Iranian children and adolescents: the CASPIAN studies

**DOI:** 10.1186/s12889-022-13045-8

**Published:** 2022-03-29

**Authors:** Mohammad Reza Hashemi-Aghdam, Gita Shafiee, Mehdi Ebrahimi, Hanieh-Sadat Ejtahed, Mehdi Yaseri, Mohammad Esmaeil Motlagh, Mostafa Qorbani, Ramin Heshmat, Roya Kelishadi

**Affiliations:** 1 Chronic Diseases Research Center, Endocrinology and Metabolism Population Sciences Institute, Tehran University of Medical Sciences, Tehran, Iran; 2 Department of Internal Medicine, Sina Hospital, Tehran University of Medical Sciences, Tehran, Iran; 3 Obesity and Eating Habits Research Center, Endocrinology and Metabolism Clinical Sciences Institute, Tehran University of Medical Sciences, Tehran, Iran; 4 Endocrinology and Metabolism Research Center, Endocrinology and Metabolism Clinical Sciences Institute, Tehran University of Medical Sciences, Tehran, Iran; 5 Department of Epidemiology and Biostatistics, School of Public Health, Tehran University of Medical Sciences, Tehran, Iran; 6 Department of Pediatrics, Ahvaz Jundishapur University of Medical Sciences, Ahvaz, Iran; 7 Non-communicable Diseases Research Center, Alborz University of Medical Sciences, Karaj, Iran; 8 Department of Pediatrics, Child Growth and Development Research Center, Research Institute for Primordial Prevention of Non-communicable Disease, Isfahan University of Medical Sciences, Isfahan, Iran

**Keywords:** Passive smoking, Trend, Adolescents, Children

## Abstract

**Background:**

It is well documented that, similar to active smokers, passive smokers are also at an increased risk of developing non-communicable diseases, and it could impose high financial costs on the healthcare system. This study aimed to evaluate the trend of passive smoking and related determinants during the three phases of a school-based surveillance program.

**Methods:**

This is a secondary study using the national data obtained from three phases of the surveillance program entitled The Childhood and Adolescence Surveillance and Prevention of Adult Noncommunicable Disease (CASPIAN) study, conducted from 2008 to 2014 on Iranian children and adolescents living in urban and rural areas of 30 provinces in Iran. Participants were selected by cluster multistage sampling method.

**Results:**

Overall, the study participants consisted of 33,288 students (50.5% boys) with a mean (± SD) age of 12.8 ± 3.2 years. The passive smoking rate was significantly increased from 35.6% in 2008 to 43.2% in 2015 among children and adolescents. According to the multivariate logistic regression, father’s university education, mother’s employment, life satisfaction, and socioeconomic status had a protective role regarding second-hand smoke exposure. In contrast, the father’s self-employment had a positive role in increasing the rate of passive smoking.

**Conclusion:**

Considering the increasing trend of passive smoking and its considerable adverse health effects, it is necessary to implement large-scale public interventions to reduce the rate and hazards of exposure to tobacco smoke.

## Background

Passive smoking refers to the state in which an individual is involuntarily exposed to the smoke from other peoples’ cigarettes, hookah, and other tobacco substances. Studies show that similar to active smokers, passive smokers are also at an increased risk of developing cardiovascular diseases [1], upper and lower respiratory tract infections [2] ,pulmonary diseases [3], and lung cancer [4]. Moreover, exposure to second-hand smoke (SHS) increases the mortality rate in chronic obstructive pulmonary disease (COPD) patients [5]. In 2004, 603,000 deaths were attributable to SHS, which consisted of about 1% of worldwide mortality in the same year. Notably, disability-adjusted life years (DALY) due to smoking was 10.9 million years in the same year. Exposure to SHS increases the risk of lower respiratory tract infection in children< 5 years, asthma in adults and children, ischemic heart diseases, and lung cancer in adults, which had the most significant burden of diseases [6]. Women bear nearly 80% of the total passive smoking burden [7]. Furthermore, evidence also shows a cause-and-effect relationship between passive smoking and sudden infant death syndrome (SIDS) [8, 9]. For every 1% increase in smoking-free houses in the US, a 0.4% decrease in the SIDS rate was observed from 1995 to 2006 [10].

Cigarette smoking has an immunosuppressive effect [11]; as a result, leukocyte dysfunction is found in children with smoker parents. Therefore, children exposed to cigarette smoke are at a higher risk of common cold, asthma, Otitis Media (OM), and respiratory complications like bronchitis and pneumonia, and thus they endure longer absentees at school [9, 12, 13]. Furthermore, smoke exposure during pregnancy is associated with the increasing prevalence of “physician-diagnosed asthma” in the child [14].

Children growing up with smoking parents or friends have a higher chance of turning into active smokers in the future [15–17]. Moreover, neurological and neuro-behavioural developmental defects are higher in passive smoking children as there is a 50% higher chance of developing at least two neuro-behavioural disorders, including autistic disorders, Attention deficit hyperactivity disorder (ADHD), and disruptive behaviour disorder in passive smoking children compared to others [18–20].

Children experiencing long-term exposure to SHS have impaired pulmonary evolution and will never reach their maximum pulmonary capacity [21, 22], and the incidence rate of lung and upper respiratory tract cancers is higher in these children [23, 24]. Furthermore, long-term exposure to SHS in children increases their future risk of developing cardiovascular diseases. Cardiovascular risk factors, including obesity, overweight, low high-density lipoprotein cholesterol (HDL-C), and high low-density lipoprotein cholesterol (LDL-C), are more prevalent in children exposed to SHS [25, 26]. In adolescents, passive smoking is also independently associated with metabolic syndrome [27]. In China, 69% of young women reported that they are exposed to SHS, and 49.9% are exposed to SHS on a daily basis [28]. Moreover, studies have shown SHS exposures as high as 32% in Iranian infants [29]. In other studies in Iran, the reported SHS exposure in 13 to 15-year-olds adolescents was considerable [30]. A recent meta-analysis showed that exposure to SHS in Iran was increased and mostly happened in the houses (as high as 49.7% in women and 54.8 in men) [31].

However, despite all evaluations, there is no evidence regarding the trends of passive smoking in Iranian children and adolescents throughout recent years. Moreover, these studies have been conducted in different geographical regions with different design. Considering that exposure to SHS has significant and even long-term consequences on children and adolescents’ health and imposes considerable costs on healthcare system, the present study aimed to evaluate the trend of passive smoking during phases 3, 4, and 5 of Childhood and Adolescence Surveillance and Prevention of Adult Noncommunicable Disease (CASPIAN) study (2008–2014) in Iranian children and adolescents and find the determining factors. Furthermore, performing trend studies is essential to evaluate risk factors changes during time, and it could be useful for health policymakers.

## Methods

This study is a second-hand data analysis aligned with Iranian national studies entitled “Childhood and Adolescence Surveillance and Prevention of Adult Non-communicable Disease (CASPIAN)”, which gathered and analyzed the data obtained from CASPIAN-III, IV, and V phases. The methodology of these surveys was published previously in detail [32–34].

The sample size in each survey was calculated based on the cluster sampling method to achieve an acceptable estimate of the main risk factors of interest. Briefly, CASPIAN-III [32] was conducted among 5570 students (10–18 years) in 2009–2010. CASPIAN-IV [33] was carried out among 14,880 students aged 7–18 years in 2011–2012, and CASPIAN-V [34] was performed among 14,400 students aged 7–18 years in 2014–2015. Five thousand five hundred twenty-eight students in CASPIAN-III, 13,486 subjects in CASPIAN-IV, and 14,274 participants in CASPIAN-V had complete data, so the total number of 33,288 participants with complete data entered the study.

The sampling method in all three phases was multistage cluster sampling conducted in urban and rural areas across the country. Sampling was performed in proportion with the number of students in each residential area and school level with an equal sex ratio; i.e., the number of male and female students was equal in each province, and the ratio of urban/rural students of every province was proportionate to the total urban/rural student population. Cluster sampling with equal clusters was used to reach the necessary sample size in each province. Clusters were determined at the level of schools, including 10 sample units (students and their parents) in each cluster. The study’s protocol was approved by the ethical committee of Alborz University of Medical Sciences, and all methods were carried out in accordance with relevant guidelines and regulations. Informed written and verbal consents from the parents and students were obtained following an explanation of the aims and procedure of the study.

### Questionnaires

Two specific questionnaires were considered for students and their parents. The students’ questionnaire was obtained from the World Health Organization-Global School Student Health Survey (WHO-GSHS) translated into Persian. The validity and reliability of questionnaires have been assessed previously [35].

The questionnaire comprised different sections, including friends’ relationships, students’ schools, life satisfaction, health behaviours, physical activity, and leisure time activities. Parents of students were also invited to complete the parent’s questionnaire regarding the family’s socioeconomic status, educational level, job, and health-related behaviours.

According to the GHSH questionnaire, students were asked to report whether their family members (father, mother, siblings, other) used tobacco products (cigarette and hookah smoking, etc.) in their presents. If positive, the student was considered as an SHS.

In order to evaluate screen time (ST) behaviours, the number of hours spent on watching television (TV), video, computer, or playing video games were asked, and the total hours were calculated as ST. Using a reliable questionnaire, weekly leisure-time physical activity data were collected. At least 30 min of daily exercise led to sweating, and a significant increase in heartbeat or breathing was considered as sufficient physical activity. Based on previously defined criteria, in addition to the familial level of SES, we considered the regional level of SES as well. Iran was classified into four sub-national regions using principal component analysis. The regions include Central, Western, North-Northeast, and the Southeast regions ordered from high to low SES, respectively [36].

### Statistical analysis

STATA package ver. 11.0 (Stata Statistical Software: Release 11. College Station, TX: Stata Corp LP. Package) was used for statistical analysis. Quantitative data were reported as mean ± SD, and qualitative data were expressed as numbers (percentage). The Chi-square test was used to compare qualitative variables among groups. Considering the dual purpose of this study, i.e., evaluating the trend and determinants of passive smoking, the trend analysis was performed on the total data; then, the trend was studied according to each independent variable. The trend of SHS according to independent variables was assessed using the Chi-Square test for trend. Moreover, evaluating the determinants of passive smoking was done on the total data. In order to evaluate passive smoking determinants, each independent variable was initially entered into the univariate logistic regression model. Then, variables with a *P*-Value < 0.20 were entered in the multivariate logistic regression model. The logistic regression model results were presented as odds ratio (OR) and 95% confidence interval (CI). A *P*-Value < 0.05 was considered statistically significant.

## Results

The mean (SD) of the age of the participants was 12.8 ± 3.2 years. 27.5, 38.5, and 33.9% of the students were in 7–10, 11–14, and 15–18 years age groups, respectively. 50.5% of participants were boys, and 49.5% were girls. The percentage of urban and rural students was 72.8 and 27.2%, respectively. In CASPIAN-III, students aged 10 to 18 years were studied; therefore, there is no data regarding 7–10 years old students. The demographic characteristics of the participants are demonstrated in Table 1. The total passive smoking rate in the present study was 42.2%. Table 2 shows the prevalence of passive smoking according to demographic characteristics and surveys can be seen. The distribution of second-hand smoke exposure was significantly different according to age groups, fathers’ and mothers’ education and occupation, life satisfaction, socioeconomic status, screen time, and physical activity.

**Table 1:** Characteristics of participants according to the different phases of CASPIAN studies

Demographic information	CASPIAN-III (2009–2010)	CASPIAN-IV (2011–2012)	CASPIAN-V (2014–2015)	*P*-value
Age				
7–10 years	**(−)**	4349 (32.2)	4843 (33.9)	< 0.001*
11–14 years	2593 (46.1)	4678 (34.7)	5591 (39.2)	
15–18 years	3032 (53.9)	4459 (33.1)	3840 (26.9)	
Gender				
Boy	2801 (49.8)	6846 (50.8)	7228 (50.6)	0.45
Girl	2824 (50.2)	6640 (49.2)	7046 (49.4)	
Place of residence				
Urban	3785 (69.3)	10,191 (75.6)	10,194 (71.4)	< 0.001
Rural	1677 (30.7)	3295 (24.4)	4080 (28.6)	
Father’s occupation				
Unemployed	342 (6.4)	657 (5.0)	860 (6.2)	< 0.001
Worker/Employee	3077 (57.2)	7309 (56.1)	8110 (58.8)	
Self-employed	1961 (36.4)	5062 (38.9)	4833 (35.0)	
Mother’s occupation				
Housewife	5002 (91.0)	11,883 (89.0)	12,354 (87.2)	< 0.001
Worker/Employee	386 (7.0)	1060 (7.9)	1300 (9.2)	
Other	110 (2.0)	409 (3.1)	514 (3.6)	
Father’s education				
Illiterate	807 (14.8)	1471 (11.2)	1734 (12.6)	< 0.001
High school diploma or lower	4125 (75.7)	9788 (74.8)	10,163 (73.7)	
Bachelor degree or higher	520 (9.5)	1831 (14.0)	1893 (13.7)	
Mother’s education				
Illiterate	1263 (22.9)	2270 (17.0)	2500 (17.6)	< 0.001
High school diploma or lower	3973 (72.2)	9925 (74.3)	10,148 (71.6)	
Bachelor degree or higher	269 (4.9)	1167 (8.7)	1524 (10.8)	
Family members				
4 or less	1953 (39.4)	6491 (48.9)	6742 (47.9)	< 0.001
5 and more	3008 (60.6)	6778 (51.1)	7336 (52.1)	
Life satisfaction				
Satisfied	3135 (56.9)	10,698 (79.9)	11,216 (78.9)	< 0.001
Dissatisfied	2379 (43.1)	2689 (20.1)	2996 (21.1)	
Socio-economic status of family				
Weak	1052 (19.8)	4147 (33.5)	4562 (33.5)	< 0.001
Moderate	2085 (39.2)	4100 (33.1)	4521 (33.2)	
Good	2178 (41.0)	4143 (33.4)	4555 (33.4)	
Socio-economic status of place of residence				
South east	557 (9.9)	1181 (8.8)	1919 (13.4)	< 0.001
North & north east	1073 (19.1)	2359 (17.5)	2398 (16.8)	
West	2340 (41.6)	6119 (45.4)	6597 (46.2)	
Central	1654 (29.4)	3827 (28.4)	3360 (23.5)	
Physical activity				
Light	2293 (41.5)	4553 (34.1)	4454 (33.4)	< 0.001
Moderate	1818 (32.9)	4910 (36.8)	4424 (33.2)	
Intense	1417 (25.6)	3886 (29.1)	4440 (33.3)	
ST				
2 h or less	3475 (68.0)	10,899 (81.4)	12,135 (87.4)	< 0.001
More than 2 h	1634 (32.0)	2494 (18.6)	1752 (12.6)	
Passive smoking				
Yes	2002 (35.6)	5802 (43.9)	5772 (43.2)	< 0.001
No	3623 (64.4)	7424 (56.1)	7575 (56.8)	

values are reported as N (%)

*CASPIAN* The Childhood and Adolescence Surveillance and Prevention of Adult Noncommunicable Disease study, *ST* screen time

*this *p*-value is for ages 11–14 and 15–18 among the CASPIAN studies

**Table 2:** The trend of passive smoking according to demographic characteristics and survey

Independent variables	All surveys *n* (%)	CASPIAN-III 2009–2010)	CASPIAN-IV (2011–2012)	CASPIAN-V (2014–2015)	*P*-trend
Participants					
13,576 (42.2)					
Age					
7–10 yrs.	3694(42.2)		41.4%	42.9%	
11–14 yrs.	5155(41.3)	35.9%	42.8%	42.7%	< 0.001
15–18 yrs.	4727(43.1)	35.3%	47.3%	44.4%	< 0.001
AM					
Boy	6805(41.8)	32.70%	44.10%	43.40%	< 0.001
Girl	6771(42.5)	38.40%	43.70%	43.10%	0.001
Place of residence					
Urban	9797(42.1)	34.4%	43.9%	43.3%	< 0.001
Rural	3714(42.4)	37.9%	43.9%	43.2%	0.003
Father’s occupation					
Unemployed	778(43.1)	35.7%	51.3%	39.8%	0.914
Worker/Employee	7294(40.9)	34.4%	41.1%	43.3%	< 0.001
Self-employed	5066(44.3)	37.0%	47.5%	44.0%	0.001
Mother’s occupation					
Housewife	12,075(42.7)	35.7%	44.4%	44%	< 0.001
Worker/Employee	876(33.7)	32.4%	34.2%	33.7%	0.754
Other	487(49.4)	39.1%	52.4%	49.4%	0.298
Father’s education					
Illiterate	1660(42.8)	37.4%	46.1%	42.4%	0.108
High school diploma or lower	10,237(44.0)	36.5%	46.3%	45.1%	< 0.001
Bachelor degree or higher	1293(31.8)	26%	30.2%	35.1%	< 0.001
Mother’s education					
Illiterate	2529(43.4)	37.9%	45.2%	44.6%	0.001
High school diploma or lower	9933(42.7)	35.3%	44.8%	43.6%	< 0.001
Bachelor degree or higher	988(35.5)	29%	33.2%	38.8%	< 0.001
Family members					
≤ 4 or less	6204(42.4)	34.8%	44.0%	43.3%	< 0.001
≥ 5 or more	6949(42.0)	35.7%	43.9%	43.1%	< 0.001
Life satisfaction					
Satisfied	9701(40.0)	27.4%	41.5%	42.3%	< 0.001
Dissatisfied	3781(48.6)	45.4%	53.1%	47.2%	0.313
Socio-economic status of family					
Weak	4195(44.6)	42.9%	47.5%	42.3%	0.013
Moderate	4540(44.0)	34.3%	46%	46.8%	< 0.001
Good	4057(38.6)	33.2%	39.3%	40.7%	< 0.001
Socio-economic status of place of residence					
South east	1371(39.6)	36.6%	39.2%	40.9%	0.068
North & north east	2176(38.5)	27.2%	39%	43.3%	< 0.001
West	6253(43.1)	38.5%	43.4%	44.6%	< 0.001
Central	3776(44.0)	36.6%	49%	41.9%	0.101
Physical activity					
Light	4605(42.9)	45.1%	45%	39.1%	< 0.001
Moderate	4456(41.1)	25.9%	43.8%	44.4%	< 0.001
Intense	3992(41.7)	32.3%	42.3%	44.1%	< 0.001
ST					
2 h or less	10,622(41.6)	33%	43.2%	42.7%	< 0.001
More than 2 h	2499(43.1)	38.9%	46.6%	42.2%	0.058

*CASPIAN* The Childhood and Adolescence Surveillance and Prevention of Adult Noncommunicable Disease study, *ST* screen time, *SHS* second-hand smoke

### The passive smoking trend during different phases of the CASPIAN study

The passive smoking rate significantly increased from 35.6% in 2008 (phase III) to 43.2% in 2015 (phase V) (*P*-Value < 0.001). The passive smoking trend is demonstrated in Table 2 according to each independent variable and their significance. Passive smoking increased among ages 11–14 and 15–18 years and both sexes. Also, an increasing trend in urban and rural areas, in people satisfied with their lives and in all family socioeconomic status sub-categories was seen. Moreover, the “North and North-east” and “West” regions of Iran had an increasing trend of passive smoking.

An increasing trend of passive smoking was observed in worker/employee and self-employed sub-categories of father’s occupation and housewife mothers. Considering parents’ education level, the passive smoking trend is increasing at all levels except illiteracy of father and all sub-categories of mother’s education.

### Evaluating the passive smoking determinants

Findings of univariate and multivariate analyses on the total data (CASPIANs III, IV and V) are presented in Table 3. Age, gender, and place of residence were not associated with SHS exposure rates in any of the models. The passive smoking rate increased when the father was self-employed (OR = 1.14, *P*-value = 0.019). Among mother’s occupation sub-categories, the worker/employee group had a protective role against SHS exposure (OR = 0.84, *P*-value = 0.03) while mother’s occupation in the “other” sub-category was a risk factor for SHS exposure (OR = 1.63, *P*-value = 0.001).

**Table 3:** Determinants of passive smoking using the univariate and multivariate logistic regression models on three phases of CASPIANs (III, IV and V)

Variables	Crude OR (%95 CI)	*P*-Value	Adjusted OR (%95 CI)	*P*-Value
Age				
7–10 yrs.	**(**Reference)	(−)	(−)	(−)
11–14 yrs.	0.97 (0.91–1.02)	0.208	(−)	(−)
15–18 yrs.	1.04 (0.98–1.10)	0.228	(−)	(−)
Gender				
Boy	**(**Reference)	(−)	(−)	(−)
Girl	1.03 (0.98–1.07)	0.247	(−)	(−)
Place of residence				
Urban	**(**Reference)	(−)	(−)	(−)
Rural	1.01 (0.97–1.07)	0.578	(−)	(−)
Father’s occupation				
Unemployed	**(**Reference)	(−)	**(**Reference)	(−)
Worker/Employee	0.91 (0.83–1.01)	0.066	1.04 (0.93–1.16)	0.475
Self-employed	1.05 (0.95–1.16)	0.342	1.14 (1.02–1.28)	0.019
Mother’s occupation				
Housewife	**(**Reference)	(−)	**(**Reference)	(−)
Worker/Employee	0.68 (0.63–0.74)	< 0.001	0.84 (0.75–0.94)	0.003
Other	1.31 (1.15–1.49)	< 0.001	1.63 (1.41–1.88)	< 0.001
Father’s education				
Illiterate	**(**Reference)	(−)	**(**Reference)	(−)
High school diploma or lower	1.05 (0.98–1.13)	0.139	1.07 (0.98–1.17)	0.135
Bachelor degree or higher	0.62 (0.57–0.68)	< 0.001	0.73 (0.65–0.83)	< 0.001
Mother’s education				
Illiterate	**(**Reference)	(−)	**(**Reference)	(−)
High school diploma or lower	0.97 (0.92–1.03)	0.344	1.05 (0.97–1.16)	0.221
Bachelor degree or higher	0.72 (0.65–0.79)	< 0.001	1.10 (0.96–1.26)	0.173
Family members				
≤ 4 or less	**(**Reference)	(−)	(−)	(−)
≥ 5 or more	0.98 (0.94–1.03)	0.478	(−)	(−)
Life satisfaction				
Dissatisfied	**(**Reference)	(−)	**(**Reference)	(−)
Satisfied	0.70 (0.67–0.74)	< 0.001	0.71 (0.67–0.75)	< 0.001
Socioeconomic status of family				
Weak	**(**Reference)	(−)	**(**Reference)	(−)
Moderate	0.98 (0.92–1.03)	0.382	0.92 (0.86–0.98)	0.015
Good	0.78 (0.74–0.83)	< 0.001	0.83 (0.77–0.89)	< 0.001
Socioeconomic status of place of residence				
Southeast	**(**Reference)	(−)	**(**Reference)	(−)
North & north east	0.95 (0.87–1.04)	0.264	1.02 (0.93–1.12)	0.714
West	1.15 (1.07–1.24)	< 0.001	1.22 (1.12–1.32)	< 0.001
Central	1.20 (1.11–1.30)	< 0.001	1.31 (1.20–1.44)	< 0.001
Physical activity				
Light	**(**Reference)	(−)	**(**Reference)	(−)
Moderate	0.93 (0.88–0.98)	0.008	0.95 (0.90–1.01)	0.110
Intense	0.95 (0.90–1.01)	0.085	1.02 (0.96–1.09)	0.461
ST				
2 h or less	**(**Reference)	(−)	**(**Reference)	(−)
More than 2 h	1.06 (1.00–1.13)	0.037	1.00 (0.94–1.06)	0.913

*OR* odds ratio, *CI* confidence interval, *ST* screen time

Fathers with university education had a protective role against SHS exposure (OR = 0.73, *P*-value < 0.001). However, the mother’s education did not play a role in SHS exposure in the multivariate regression model.

Satisfaction with life had a protective role against SHS exposure compared to dissatisfaction (OR = 0.71, *P*-value < 0.001). Moreover, a moderate or good socio-economic status of family decreased passive smoking rates (OR = 0.72, *P*-value = 0.015 and OR = 0.83, *P*-value < 0.001; respectively). Residing in Iran’s west and central socio-economic regions increased the passive smoking rates (OR = 1.22, *P*-value < 0.001; and OR = 1.31, *P*-value < 0.001; respectively). Unlike the univariate logistic regression model, physical activity or ST was not associated with SHS exposure in the multivariate regression model.

## Discussion

In Iran, passive smoking has had an increasing trend among Iranian children and adolescents in different study phases and has increased significantly from 35.6% of students in 2008 (phase III) to 43.2% of participants in 2015 (phase V). There was no association between passive smoking, age groups and gender in this study. According to our multivariate logistic regression model results, the fathers’ academic education was associated with decreased passive smoking. However, the mothers’ education was not associated with passive smoking exposure in their children. Moreover, being a self-employed father was a risk factor that increased passive smoking exposure rates in children and adolescents. Nonetheless, the mother’s employee/worker occupation had a protective role while the “other” category of the mother’s occupation was a risk factor for passive smoking exposure in their children.

Regarding the trend of passive smoking, contrary to Iran, studies report a decreasing trend in both sexes in many countries. Passive smoking had a decreasing trend in Germany in different age groups in girls and boys [37]. In Vietnam, passive smoking rates in 13–15-year-old students decreased from 58.5% in 2007 to 47.1% in 2014 [38]. In UK, cotinine levels in 11–15 years-old children decreased from 0.96 ng/ml in 1988 to 0.52 ng/ml in 1998 [39]. Furthermore, serum cotinine levels of 4–15 years-old children decreased from 0.52 ng/ml in 1998 to 0.11 ng/ml in 2012, indicating a significant decrease in SHS exposure during these years [40]. In Finland, SHS exposure in adolescents decreased from 17% in 1991 to 6% in 2009 [41]. Local and regional studies indicate a high and increasing exposure to SHS in Iran [31]. The increasing trend of passive smoking in Iran may be due to the lack of parents’ education regarding the adverse health complications of SHS in children/adolescents and the weaknesses in enforcing the Law in smoke-free environments, and the ease of acquiring tobacco even in those who are under the legal age (18 years). One other important factor is the popularity of hookah among the Iranian population, as many restaurants and cafes serve hookah, exposing all the customers to SHS [31].

Our results were in line with some studies regarding the association of passive smoking and age groups. In a study conducted in the Global Youth Tobacco Survey (GYTS) framework in South Africa in 2008, no association was found between age and exposure to smoking [42]. In another study conducted on secondary and high school students in the US, there was no association between age groups and SHS exposure [43]. On the contrary, some other studies reported a positive association between passive smoking and age. Studies conducted in India, Gambia, and Malaysia showed a positive association between exposure to smoking both inside and outside of the home and older age [44–46]. On the other hand, the findings of some studies indicated that younger children were at greater risk for passive smoking [47, 48]. Our results can be due to the use of tobacco substances in public places, restaurants, cafes, and parks in which most adolescents hangout, as well as, due to the popularity of hookahs, especially among adolescents, and its ease of use in the public and gatherings without restrictions [31, 43].

Contradictory to our study’s findings, other studies evaluating the relationship between gender and passive smoking showed higher exposure rates to passive smoking in girls [43, 44, 47], while in some other studies, boys were exposed to higher rates of passive smoking [46]. Nonetheless, similar to our findings, Some other studies did not report any relationship between gender and passive smoking [42, 48]. Our findings could be the results of cultural differences among different countries, as in Iran, many households possess hooka and use it regularly, condemning all households, regardless of age and gender, to SHS [31].

Our finding of the multivariate logistic regression model was in line with some other studies. A study performed in Granada, Spain (1999) showed that lower parent’s awareness of smoking at home and low fathers’ education level are associated with higher urinary cotinine levels [49]. Moreover, in a study in Malaysia (2009), salivary cotinine levels were lower in students with university-educated fathers than fathers with a high school diploma or lower levels of education [50]. Furthermore, in a study conducted in Korea (2012), lower education levels of fathers were associated with higher passive smoking rates [51]. This finding may be due to the fact that fathers with university education are probably more aware of the health complications of smoking exposure in their children and avoid smoking in their presence. However, the mother’s education had an inverse association with passive smoking [38, 52, 53]. It should be noted that some studies did not demonstrate any relationship between a mother’s education level and passive smoking exposure in children [49, 51, 54]. R.egarding parental occupation, our results were similar to other studies. For example, in a study performed in Malaysia (2009), salivary cotinine levels in students with fathers having military jobs were higher than those whose fathers had management/professional occupations [50]. The explanation could be that fathers with higher education levels were not usually self-employed. Moreover, most families with professional and management occupations fall within higher SES groups, and exposure to SHS is lower within this group.

The present study determined that students satisfied with their lives are less exposed to passive smoking; this observation was also reported in other studies. Results of the CASPIAN IV showed passive smoking exposure was associated with lower psychological health, higher violence, anxiety, stress and depression, and inappropriate living conditions of children and adolescents [55], which could lead to a reduction in life satisfaction [56]. As it seems that bad SES can be related to lower life satisfaction. This finding can be the result of the association of SES and SHS [56, 57]. As those with lower life satisfaction and mental distress are at an increased risk of active smoking and being exposed to SHS as well [56].

In this study, students with good socioeconomic levels experienced lower passive smoking exposure than those with moderate/low SES levels. This finding aligns with other studies demonstrating the association between low socioeconomic levels and passive smoking exposure in children. In a study conducted on 4–15 years-old children in the UK (1996–2006), the low socioeconomic level was associated with much more passive smoking exposure [58]. Furthermore, in a study on children and adolescents older than 12 years in Australia (2010), high socioeconomic level was negatively correlated with passive smoking exposure, similar in urban and rural areas [59]. On the contrary, the multivariate logistic regression model findings showed that students residing in the west and central socioeconomic regions (higher socioeconomic regions of Iran) had higher rates of passive smoking exposure. This association was also confirmed in the Korean society [51]. This finding can be the result of the usage of hookahs in cafes and restaurants, and higher frequency of these places within high SES regions and their popularity among the residing population [31].

### Limitations and strengths

The large sample size of the present study, which was taken from different urban and rural areas of Iran, could be representative of the Iranian children and adolescents population. Therefore, it is possible to generalize the findings to society. This study has some limitations too. First, the effect of unknown confounders on the results should be addressed. Moreover, due to the CASPIAN study’s cross-sectional nature, the cause and effect relationship could not distinguish.

## Conclusion

Considering the increasing trend of passive smoking and its considerable adverse health effects, it is necessary to implement large-scale public interventions to reduce the rate and hazards of exposure to tobacco smoke. Extra taxes on tobacco and fines for public smoking can be beneficial. It should be kept in mind that educating the parents through school meetings, social media, and television programs could significantly affect passive smoking trends. Further studies are needed to investigate the social and cultural factors that contributed to this rising trend and the consequences of this increase, on the prevalence of non-communicable diseases.

## Data Availability

The datasets used and/or analyzed during the current study are available from the corresponding author on reasonable request.

## Abbreviations

ADHD: Attention Deficit Hyperactivity Disorder
BMI: Body Mass Index
CASPIAN: Childhood and Adolescence Surveillance and Prevention of Adult Non-communicable Disease
CDC: Centers for Disease Control
COPD: Chronic Obstructive Pulmonary Disease
CRP: C-Reactive Protein
DALY: Disability Adjusted Life Year
FEF 2575: Forced Expiratory Flow in 25%75%
FEV1: Forced Expiratory Volume in One score
FEV1/FVC: Forced Expiratory Volume in One score/Forced Vital Capacity
GATS: Global Adult Tobacco Survey
GYTS: Global Youth Tobacco Survey
HDLC: High-Density Lipoprotein Cholesterol
IHD: Ischemic Heart Disease
IL6: Interleukin 6
KiGGS: German Health Interview and Examination Survey for Children and Adolescents
LDLC: Low Density Lipoprotein Cholesterol
LRI: Lower Respiratory Infection
*NHANES*: *National Health and Nutrition Examination Survey*
OM: Otitis Media
RCT: Randomized Controlled Trials
SHS: Second- Hand Smoke
SHSe: Second- Hand Smoke exposure
SIDS: Sudden Infant Death Syndrome
WHO-GSHS: World Health Organization-Global school-based Student Health Survey

## References

[1] Bonita R, Duncan J, Truelsen T, Jackson RT, Beaglehole R (1999). Passive smoking as well as active smoking increases the risk of acute stroke. Tob Control.

[2] Gryczynska D, Kobos J, Zakrzewska A (1999). Relationship between passive smoking, recurrent respiratory tract infections and otitis media in children. Int J Pediatr Otorhinolaryngol.

[3] Hagstad S, Bjerg A, Ekerljung L, Backman H, Lindberg A, Ronmark E (2014). Passive smoking exposure is associated with increased risk of COPD in never smokers. Chest..

[4] Brownson RC, Alavanja MC, Hock ET, Loy TS (1992). Passive smoking and lung cancer in nonsmoking women. Am J Public Health.

[5] Ukawa S, Tamakoshi A, Yatsuya H, Yamagishi K, Ando M, Iso H (2017). Passive smoking and chronic obstructive pulmonary disease mortality: findings from the Japan collaborative cohort study. Int J Public Health.

[6] Oberg M, Jaakkola MS, Woodward A, Peruga A, Pruss-Ustun A (2011). Worldwide burden of disease from exposure to second-hand smoke: a retrospective analysis of data from 192 countries. Lancet (London, England).

[7] Cai L, Cui W, He J, Wu X (2014). The economic burden of smoking and second-hand smoke exposure in rural south-West China. J Asthma.

[8] Anderson HR, Cook DG (1997). Passive smoking and sudden infant death syndrome: review of the epidemiological evidence. Thorax..

[9] Dybing E, Sanner T (1999). Passive smoking, sudden infant death syndrome (SIDS) and childhood infections. Hum Exp Toxicol.

[10] Behm I, Kabir Z, Connolly GN, Alpert HR (2012). Increasing prevalence of smoke-free homes and decreasing rates of sudden infant death syndrome in the United States: an ecological association study. Tob Control.

[11] Qiu F, Liang CL, Liu H, Zeng YQ, Hou S, Huang S (2017). Impacts of cigarette smoking on immune responsiveness: up and down or upside down?. Oncotarget..

[12] Strumylaite L, Kregzdyte R, Vaitkaitiene E (2005). Passive smoking and respiratory health of children. Medicina (Kaunas, Lithuania).

[13] Continente X, Arechavala T, Fernandez E, Perez-Rios M, Schiaffino A, Soriano JB (2019). Burden of respiratory disease attributable to second-hand smoke exposure at home in children in Spain (2015). Prev Med.

[14] Gilliland FD, Li YF, Peters JM (2001). Effects of maternal smoking during pregnancy and environmental tobacco smoke on asthma and wheezing in children. Am J Respir Crit Care Med.

[15] Baheiraei A, Hamzehgardeshi Z, Mohammadi MR, Nedjat S, Mohammadi E (2013). Personal and family factors affecting life time cigarette smoking among adolescents in Tehran (Iran): a community based study. Oman Med J.

[16] Lampert T (2008). Smoking and passive smoking exposure in young people: results of the German health interview and examination survey for children and adolescents (KiGGS). Deutsches Arzteblatt Int.

[17] Thakur D, Gupta A, Thakur A, Mazta SR, Sharma D (2014). Prevalence of cigarette smoking and its predictors among school going adolescents of North India. South Asian J Cancer.

[18] Kabir Z, Connolly GN, Alpert HR (2011). Second-hand smoke exposure and neurobehavioral disorders among children in the United States. Pediatrics..

[19] Khalil N, Kaur B, Lawson A, Ebert J, Nahhas R (2018). Secondhand smoke exposure is associated with autism spectrum disorder in US males but not in females: Results from the National Survey on Children’s Health. Environ Dis.

[20] Bauer NS, Anand V, Carroll AE, Downs SM (2015). Second-hand smoke exposure, parental depressive symptoms and preschool behavioral outcomes. J Pediatr Nurs.

[21] Fernandez-Plata R, Rojas-Martinez R, Martinez-Briseno D, Garcia-Sancho C, Perez-Padilla R (2016). Effect of Passive Smoking on the Growth of Pulmonary Function and Respiratory Symptoms in Schoolchildren. Rev Investig Clin.

[22] Lebowitz MD, Sherrill D, Holberg CJ (1992). Effects of passive smoking on lung growth in children. Pediatr Pulmonol.

[23] Asomaning K, Miller DP, Liu G, Wain JC, Lynch TJ, Su L (2008). Second hand smoke, age of exposure and lung cancer risk. Lung Cancer.

[24] Troy JD, Grandis JR, Youk AO, Diergaarde B, Romkes M, Weissfeld JL (2013). Childhood passive smoke exposure is associated with adult head and neck cancer. Cancer Epidemiol.

[25] Kelishadi R, Noori A, Qorbani M, Rahimzadeh S, Djalalinia S, Shafiee G (2016). Are active and passive smoking associated with cardiometabolic risk factors in adolescents? The CASPIAN-III study. Paediatr Int Child Health.

[26] Neufeld EJ, Mietus-Snyder M, Beiser AS, Baker AL, Newburger JW (1997). Passive cigarette smoking and reduced HDL cholesterol levels in children with high-risk lipid profiles. Circulation..

[27] Weitzman M, Cook S, Auinger P, Florin TA, Daniels S, Nguyen M (2005). Tobacco smoke exposure is associated with the metabolic syndrome in adolescents. Circulation..

[28] Nan X, Lu H, Wu J, Xue M, Guo W, Wang X (2020). Prevalence, knowledge and education level associated with second-hand smoke exposure among never-smoking women in Inner Mongolia, northern China. Tob Induc Dis.

[29] Shiva F, Shamshiri AR, Ghotbi F, Yavari SF (2011). Exposure to secondhand smoke in infants: declining trends from 2001 to 2008?. Asia Pac J Public Health.

[30] Kashani H, Nakhjirgan P, Hassanvand MS, Shamsipour M, Yunesian M, Farzadfar F, Naddafi K, Mesdaghinia A (2021). Subnational exposure to second-hand smoke in Iran from 1990 to 2013: a systematic review. Environ Sci Pollut Res Int.

[31] Janjani H, Nabizadeh R, Kashani H, Shamsipour M, Aghaei M, Yunesian M (2021). Spatiotemporal variability of exposure to second-hand smoke in Iran during 2009-2020: a systematic review. Environ Sci Pollut Res Int.

[32] Kelishadi R, Heshmat R, Motlagh ME, Majdzadeh R, Keramatian K, Qorbani M, Taslimi M, Aminaee T, Ardalan G, Poursafa P, Larijani B (2012). Methodology and Early Findings of the Third Survey of CASPIAN Study: A National School-based Surveillance of Students' High Risk Behaviors. Int J Prev Med.

[33] Kelishadi R, Ardalan G, Qorbani M, Ataie-Jafari A, Bahreynian M, Taslimi M, Motlagh ME, Heshmat R (2013). Methodology and Early Findings of the Fourth Survey of Childhood and Adolescence Surveillance and Prevention of Adult Non-Communicable Disease in Iran: The CASPIAN-IV Study. Int J Prev Med.

[34] Motlagh ME, Ziaodini H, Qorbani M, Taheri M, Aminaei T, Goodarzi A, Ataie-Jafari A, Rezaei F, Ahadi Z, Shafiee G, Shahsavari A, Heshmat R, Kelishadi R (2017). Methodology and early findings of the fifth survey of childhood and adolescence surveillance and prevention of adult noncommunicable disease: the CASPIAN-V study. Int J Prev Med.

[35] Kelishadi R, Majdzadeh R, Motlagh ME, Heshmat R, Aminaee T, Ardalan G (2012). Development and evaluation of a questionnaire for assessment of determinants of weight disorders among children and adolescents: the Caspian-IV study. Int J Prev Med.

[36] Farzadfar F, Danaei G, Namdaritabar H, Rajaratnam JK, Marcus JR, Khosravi A (2011). National and subnational mortality effects of metabolic risk factors and smoking in Iran: a comparative risk assessment. Popul Health Metrics.

[37] Kuntz B, Lampert T (2016). Smoking and passive smoke exposure among adolescents in Germany. Deutsches Arzteblatt Int.

[38] Lam NT, Nga PT, Minh HV, Giang KB, Hai PT, Huyen DT (2016). Trends in second-hand tobacco smoke exposure levels at home among Viet Nam school children aged 13-15 and associated factors. Asian Pac J Cancer Prev.

[39] Jarvis MJ, Goddard E, Higgins V, Feyerabend C, Bryant A, Cook DG (2000). Children's exposure to passive smoking in England since the 1980s: cotinine evidence from population surveys. BMJ.

[40] Jarvis MJ, Feyerabend C (2015). Recent trends in children's exposure to second-hand smoke in England: cotinine evidence from the health survey for England. Addiction.

[41] Raisamo SU, Doku DT, Heloma A, Rimpela AH (2014). Persistence of socioeconomic differences in adolescents' environmental tobacco smoke exposure in Finland: 1991-2009. Scand J Public Health.

[42] Peltzer K (2011). Determinants of exposure to second-hand tobacco smoke (SHS) among current non-smoking in-school adolescents (aged 11-18 years) in South Africa: results from the 2008 GYTS study. Int J Environ Res Public Health.

[43] Agaku IT, Singh T, Rolle I, Olalekan A-Y, King BA (2016). Prevalence and determinants of secondhand smoke exposure among middle and high school students. Pediatrics..

[44] Raute LJ, Pednekar MS, Mistry R, Gupta PC, Pimple SA, Shastri SS (2012). Determinants of exposure to second-hand smoke at home and outside the home among students aged 11-17 years: results from the Mumbai student tobacco survey 2010. Indian J Cancer.

[45] Jallow IK, Britton J, Langley T (2018). Prevalence and factors associated with exposure to second-hand smoke (SHS) among young people: a cross-sectional study from the Gambia. BMJ Open.

[46] Mohd Ghazali S, Huey TC, Cheong KC, Li LH, Mohd Yusoff MF, Yusoff AF (2019). Prevalence and factors associated with second-hand smoke exposure among Malaysian adolescents. Tob Induc Dis.

[47] Bakoula CG, Kafritsa YJ, Kavadias GD, Haley NJ, Matsaniotis NS (1997). Factors modifying exposure to environmental tobacco smoke in children (Athens, Greece). Cancer Causes Contrl.

[48] Mannino DM, Caraballo R, Benowitz N, Repace J (2001). Predictors of cotinine levels in US children: data from the third National Health and nutrition examination survey. Chest..

[49] Jurado D, Munoz C, Luna Jde D, Fernandez-Crehuet M (2004). Environmental tobacco smoke exposure in children: parental perception of smokiness at home and other factors associated with urinary cotinine in preschool children. J Expo Anal Environ Epidemiol.

[50] Abidin EZ, Semple S, Omar A, Rahman HA, Turner SW, Ayres JG (2011). A survey of schoolchildren's exposure to second-hand smoke in Malaysia. BMC Public Health.

[51] Yi O, Kwon HJ, Kim D, Kim H, Ha M, Hong SJ (2012). Association between environmental tobacco smoke exposure of children and parental socioeconomic status: a cross-sectional study in Korea. Nicotine Tob Res.

[52] Pisinger C, Hammer-Helmich L, Andreasen AH, Jørgensen T, Glümer C (2012). Social disparities in children's exposure to second hand smoke at home: a repeated cross-sectional survey. Environ Health.

[53] Soliman S, Pollack HA, Warner KE (2004). Decrease in the prevalence of environmental tobacco smoke exposure in the home during the 1990s in families with children. Am J Public Health.

[54] Mantziou V, Vardavas CI, Kletsiou E, Priftis KN (2009). Predictors of childhood exposure to parental second-hand smoke in the house and family car. Int J Environ Res Public Health.

[55] Kelishadi R, Babaki AE, Qorbani M, Ahadi Z, Heshmat R, Motlagh ME (2015). Joint Association of Active and Passive Smoking with psychiatric distress and violence behaviors in a representative sample of Iranian children and adolescents: the CASPIAN-IV study. Int J Behav Med.

[56] Heshmat R, Qorbani M, Safiri S, Eslami-Shahr Babaki A, Matin N, Motamed-Gorji N (2017). Association of passive and active smoking with self-rated health and life satisfaction in Iranian children and adolescents: the CASPIAN IV study. BMJ Open.

[57] Chen W, Niu GF, Zhang DJ, Fan CY, Tian Y, Zhou ZK (2016). Socioeconomic status and life satisfaction in Chinese adolescents: Analysis of self-esteem as a mediator and optimism as a moderator. Person Individ Differ.

[58] Sims M, Tomkins S, Judge K, Taylor G, Jarvis MJ, Gilmore A (2010). Trends in and predictors of second-hand smoke exposure indexed by cotinine in children in England from 1996 to 2006. Addiction.

[59] Longman JM, Passey ME (2013). Children, smoking households and exposure to second-hand smoke in the home in rural Australia: analysis of a national cross-sectional survey. BMJ Open.

